# Fall Risk in Elderly with Insomnia in Western Romania—A Retrospective Cross-Sectional Study

**DOI:** 10.3390/medicina59040718

**Published:** 2023-04-06

**Authors:** Marc-Dan Blajovan, Diana-Aurora Arnăutu, Daniel-Claudiu Malița, Mirela-Cleopatra Tomescu, Cosmin Faur, Sergiu-Florin Arnăutu

**Affiliations:** 1 Multidisciplinary Heart Research Center, Victor Babes University of Medicine and Pharmacy, 2nd Eftimie Murgu Square, 340001 Timisoara, Romania; 2 Department XV-Orthopedics—Traumatology, Urology, Radiology and Medical Imagistics, Faculty of Medicine, University of Medicine and Pharmacy “Victor Babes” Timisoara, E. Murgu Square, Nr. 2, 300041 Timisoara, Romania; 3 Timisoara County Clinical Emergency Hospital, L. Rebreanu Str., Nr. 156, 300723 Timisoara, Romania; 4 Department of Internal Medicine I, Faculty of Medicine, Victor Babes University of Medicine and Pharmacy, 2nd Eftimie Murgu Square, 340001 Timisoara, Romania; arnautu.sergiu@umft.ro; 5 Timisoara Municipal Clinical Emergency Hospital, Hector Str., Nr. 1, 300040 Timisoara, Romania

**Keywords:** elderly, fall risk, fractures, sleep disturbances, comorbidities

## Abstract

*Background and Objectives*: Falls are frequent among the elderly, imply large social and economic costs, and have serious outcomes. The purpose of this study was to investigate the links between insomnia, comorbidities, multisite pain, physical activity, and fall risk in the elderly. *Materials and Methods*: This retrospective cross-sectional study included persons recruited from nursing homes for the elderly in Timisoara. We separated the participants into two groups by the absence (group I) or presence of fractures (group II) starting with the age of 65 years. Participants were asked how they feel about their sleep using one item on a 4-point scale from the Assessment of Quality of Life questionnaire. The risk of fall was evaluated using the Falls Risk Assessment Tool. *Results*: The study enrolled 140 patients with a mean age of 78.4 ± 2.4 years (range 65–98 years), 55 of them being males (39%). By comparing the two groups, we found that the elderly with a history of fractures had a greater number of comorbidities, a higher risk of fall, and more severe sleep disturbances. When using univariate logistic regression, the occurrence of fractures in the elderly was significantly associated with the number of comorbidities, the risk of fall, and the presence of sleep disturbances (*p* < 0.0001). The multivariate regression analysis selected four independent parameters significantly linked to fractures, and these were the number of comorbidities (*p* < 0.03), the risk of fall score (*p* < 0.006), and the sleep disturbances of type 3 (*p* < 0.003) and 4 (*p* = 0.001). *Conclusions*: A fall-risk score over 14 and a number of comorbidities over 2 were notably associated with the occurrence of fractures. We also found strong positive correlations between the type of sleep disturbance and the risk of fall score, the number of comorbidities, and the number of fractures in the elderly.

## 1. Introduction

Older individuals are regarded as a vulnerable segment of the population, with an increased risk of functional degradation and health decline. According to the World Health Organization, the number of persons in this age group (65+ years old) is growing and is expected to comprise 16% of the world population by 2050, with these individuals being at increased risk of noncommunicable illnesses [1]. The Global Age Watch Index emphasizes a 96-country rating based on the number of individuals aged 60 and up. Consequently, Switzerland is ranked first, and Romania is ranked 45th, roughly in the center of this list [2]. This tendency has not been followed by a significant increase in the life expectancy of Romanian seniors. According to European Commission data, the term of pension giving in Romania is around 16.5 years. The elderly population’s living standard is precarious, emphasizing the vulnerability caused by financial insecurity, access to health care providers, decline in health status (e.g., motor limitations, mental health, and chronic diseases), and social interactions [3].

Falls are frequent among the elderly, and understanding of fall-risk factors has grown dramatically in the last decade. Falls among the elderly have a large social and economic cost and can have grave long-term outcomes; nevertheless, they can be avoided. A variety of risk factors including physiological, behavioral, and environmental variables might enhance an elderly person’s risk of falling. As a result, a multivariate, customized evaluation of fall risk is a crucial initial stage in developing and implementing effective risk-reduction programs for advanced age, and balance impairments, reduced walking speed, reduced executive functioning, lower-limb weakness, use of walking aids, fear of falling, forgetfulness bladder incontinence, and fatigue have all been identified as risk factors for falls [4,5].

Insomnia or sleep-wake disorders involve problems with the quality, timing, and amount of sleep, which result in daytime distress and impairment in functioning. Sleep-wake disorders often occur along with medical conditions or other mental health conditions such as depression, anxiety, or cognitive disorder. They are extremely common in the general population, particularly in older persons [6,7,8]. Sleep issues have been associated with a variety of negative effects, including musculoskeletal discomfort [9], memory loss [10,11], cardiovascular problems, and death [12,13]. These sleep-related health implications have a significant personal and societal impact. Several observational trials [14,15,16] that explored the links between sleep disorders and osteoporosis found inconclusive results. Recent meta-analyses, which mostly included data from cross-sectional studies, showed that short sleep duration is related with an elevated likelihood of developing osteoporosis [17,18].

However, sleep issues may raise the chance of falls and fractures, as demonstrated by several clinical studies [19].

Sleep disturbances and chronic pain syndromes are frequently comorbid, and accumulating data suggest that sleep disturbances have a greater impact upon pain than vice versa; nevertheless, few research have studied the long-term correlations between sleep disruption and pain.

The purpose of this study was to investigate the links between sleeping problems (insomnia), comorbidities, multisite pain, physical activity, and fall risk/fractures in the elderly.

## 2. Materials and Methods

### 2.1. Participants

This study was a retrospective cross-sectional study, which has a population-based cohort and was conducted in Timisoara, Romania, in March 2023. The study comprised individuals selected from nursing homes for the elderly in Timisoara, aged ≥65 years. Patients with severe dementia, those with paraparesis, those bedridden, and those with medication for anxiety/depression were excluded from the study. Participants were asked whether they had experienced a fracture at any site after the age of 65 years. We grouped the elderly into two categories in this study according to the presence or the absence of any fracture at any site starting with the age of 65 years.

At enrolment, all study participants gave written informed consent. The study was conducted in accordance with the requirements of the Helsinki Declaration and was authorized by the Ethics Committee of the “Victor Babeș” University of Medicine and Pharmacy Timișoara (nr.8/2023).

### 2.2. Measurement of Exposure

In order to assess insomnia, the participants were invited to assess their night sleep during the previous week using a 4-point score from the Assessment of Quality of Life questionnaire [20]. They selected one of the following answers regarding their type of sleep disturbance: (type 1) I am able to sleep without problems utmost of the time; (type 2) my sleep is interrupted some times, but I am able to fall back to sleep without problems; (type 3) my sleep is interrupted most nights, but I am usually able to go back to sleep without difficulty; (type 4) I sleep in short bursts only, and I am awake most of the night.

### 2.3. Measurement of Covariates

Height and weight were measured, and body mass index (BMI) (kg/m^2^) was determined. Obesity was defined as a BMI ≥ 30 kg/m^2^.

Daily physical activity was measured using a pedometer (Omron Healthcare, Kyoto, Japan) and expressed as number of steps/day. Information regarding smoking history was recorded.

Comorbidities including hypertension, established cardiovascular disease, diabetes, asthma, chronic obstructive pulmonary disease, thyroid gland disorders, osteoarthritis and rheumatoid arthritis, Parkinson’s disease, and cerebral microangiopathy were gathered from the medical reports. The number of comorbidities per subject was assessed.

Musculoskeletal tenderness was evaluated using a questionnaire with the question on whether participants had or did not have pain during the previous week in one of the following regions (neck, shoulders, backbone, hips, hands, feet, or knees). The number of painful sites was then determined. Multisite pain was stated as pain involving ≥2 sites during the same day [9].

### 2.4. Measurement of Outcomes

The risk of fall was appreciated at enrollment using the Fall Risk Assessment Tool (FRAT) [21]. The FRAT was tested with primary care practitioners, social care workers, physical therapists, and residential home caregivers. The risk of a fall for an individual was calculated based upon recent falls (none in the last 12 months, 2 points; one or more between 3 and 12 months ago, 4 points; one or more in the last 3 months, 6 points; one or more in the last 3 months whilst inpatient/resident, 8 points); medications (sedatives, anti-Parkinson’s, diuretics, anti-hypertensives, hypnotics: taking none of these—1 point; taking one—2 points; taking two—3 points; taking 3 or more—4 points); psychological problems (anxiety, depression: none of these, 1 point; mildly affected, 2 points; moderately affected, 3 points; severely affected, 4 points); cognitive status evaluated by Hodkinson Abbreviated Mental Test Score by (AMTS: AMTS 9 or 10/10 or intact, 1 point; AMTS 7–8 or mildly impaired, 2 points; AMTS 5–6 or moderately impaired, 3 points; AMTS 4 or less or severely impaired, 4 points [22].

Dual-energy X-ray absorptiometry (DXA) (Hologic Delphi W (S/N 70489) densitometer, MA, USA) was used to measure total bone mass density (BMD).

### 2.5. Statistical Analysis

MedCalc statistical software for Windows, version 20.218, was used for statistical analysis (MedCalc Software Ltd., Ostend, Belgium; https://www.medcalc.org; accessed on 14 March 2023). Chi-square tests for categorical data and Student’s *t*-tests for continuous data were applied to assess differences between study groups. Covariates considered for potential impact with fractures included age, sex, obesity, smoking status, the number of comorbidities, the daily number of steps, the presence of multisite pain, total bone mineral density (BMD), the risk of fall, the type of sleep disturbances, and the medication for sleep disturbances. For assessing the involvement of each variable in the studied outcomes, univariate and multivariate logistic regression models were established. Odds ratios (ORs) and 95% confidence intervals (CIs) were calculated using logistic regression models. Multivariate regression analysis with backward stepwise method was used for all parameters that were associated with fractures after the age of 65 years in univariate analysis. Receiver operating characteristic (ROC) curves were utilized to determine the sensitivity and specificity of the analyzed parameters. The discrimination ability of the analyzed parameters was estimated by the C-statistic, which is equivalent to the area under the ROC curve. A model with C-statistic > 0.75 is considered to have a meaningful discriminatory ability. The closer a C-statistic is to 1, the better the model is able to classify outcomes correctly. A *p*-value < 0.05 was used as the statistical significance level.

## 3. Results

The study group consisted of 140 patients with a mean age of 78.4 ± 2.4 years (range 65–98 years), and 55 were males (39%). We separated the participants into two groups by the absence (group I) or presence of fractures (group II). By comparing the two groups, we found that the elderly with a history of fractures had a greater number of comorbidities and a higher risk of fall and presented more often sleep disturbances of type 3 and 4, as shown in Table 1. The medication prescribed for insomnia was Zoplicone, a non-benzodiazepine hypnotic agent, that was significantly more often prescribed in the elderly group that suffered fractures (*p* < 0.001), as they suffered from more severe sleep disturbances. There were no statistically significant differences in age, gender, BMI, history of smoking, number of daily steps, and total bone mass density (BMD) among the two groups.

When using univariate logistic regression, the occurrence of fractures in the elderly was significantly associated with the number of comorbidities, the risk of fall, and sleep disturbances, as shown in Table 2. When moving to multivariate analysis, four independent parameters significantly linked to fractures were found, and these were the number of comorbidities, the risk of fall score, and sleep disturbances of type 3 and 4.

**Figure 1:**
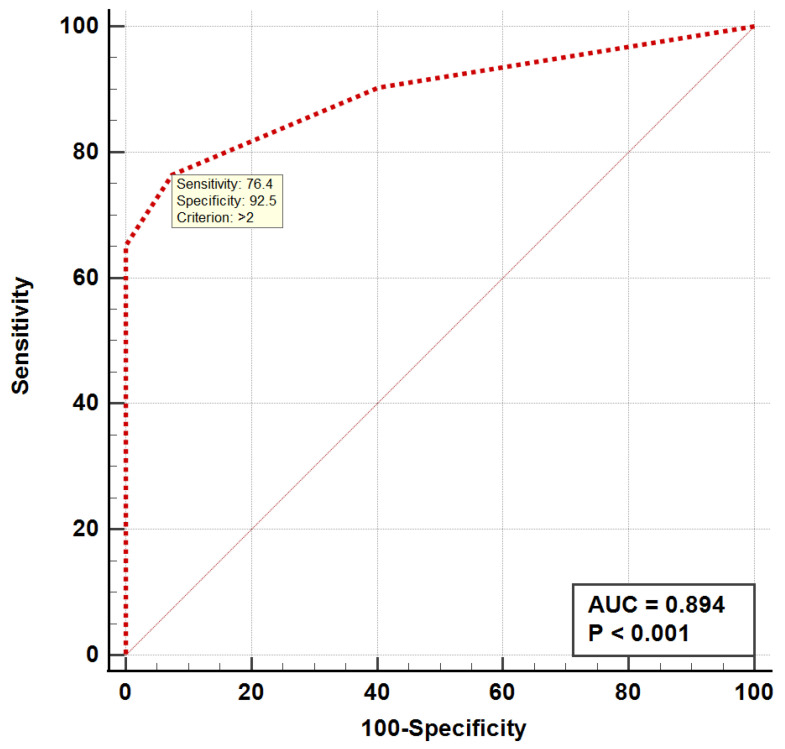
ROC curve for the number of comorbidities in the elderly with fractures. ROC, receiver operating curve; AUC, area under the curve.

**Figure 2:**
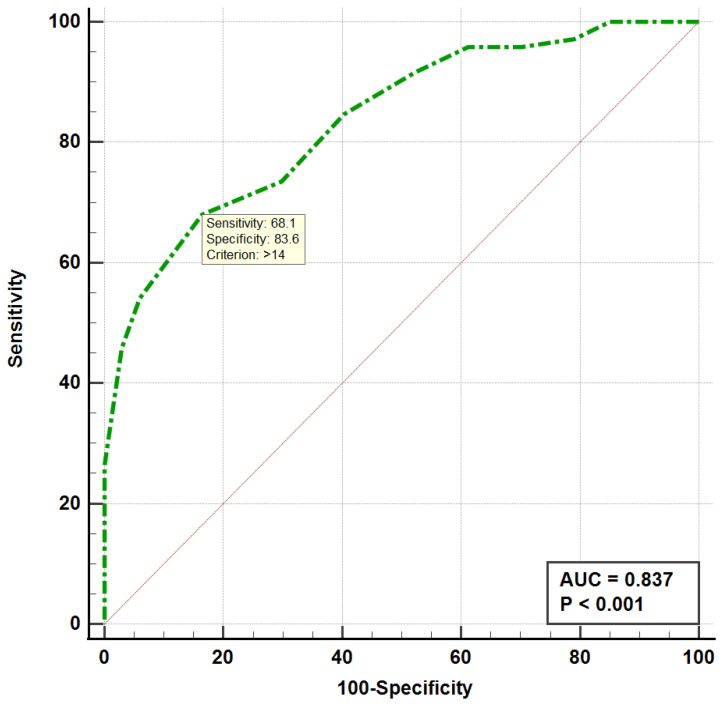
ROC curve for the risk of fall score in the elderly with fractures. ROC, receiver operating curve; AUC, area under the curve.

When analyzing the ROC curves, we found that the occurrence of fractures was significantly associated with a risk of fall score > 14 (AUC = 0.837, *p* < 0.001) and a number of comorbidities > 2 (AUC = 0.894, *p* < 0.001), as shown in Figure 1 and Figure 2. Both the ROC curve of the number of comorbidities and the ROC curve of the risk of fall showed an excellent discriminatory power regarding the occurrence of fractures in the elderly.

**Figure 3:**
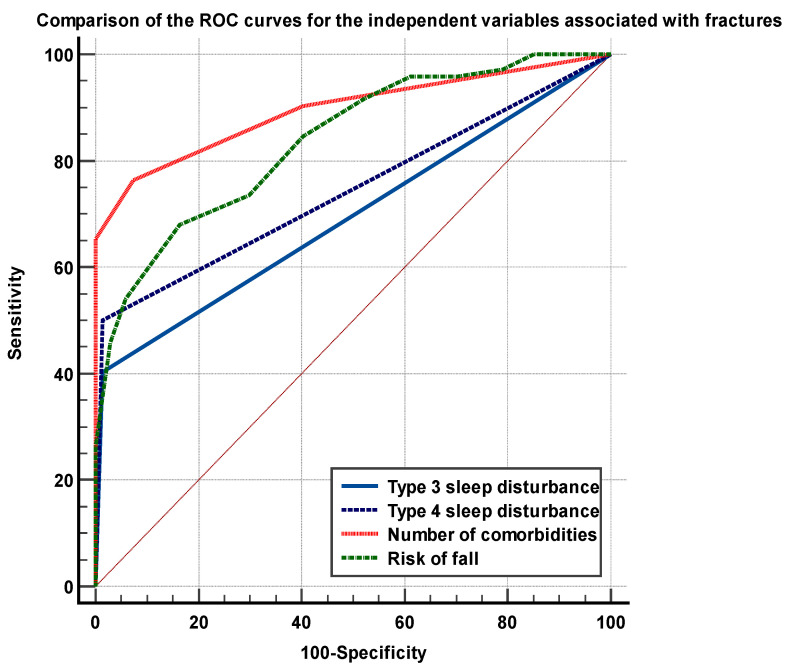
Comparison of the ROC curves of the independent parameters associated with fractures in the elderly.

The analysis of the ROC curves showed significant differences between the areas under the curves (AUC’s) of the number of comorbidities and the FRAT (having excellent discriminatory power) versus the sleep disturbances of type 3 and 4 (having reasonable discriminatory power), *p* < 0.01, as shown in Figure 3.

**Figure 4:**
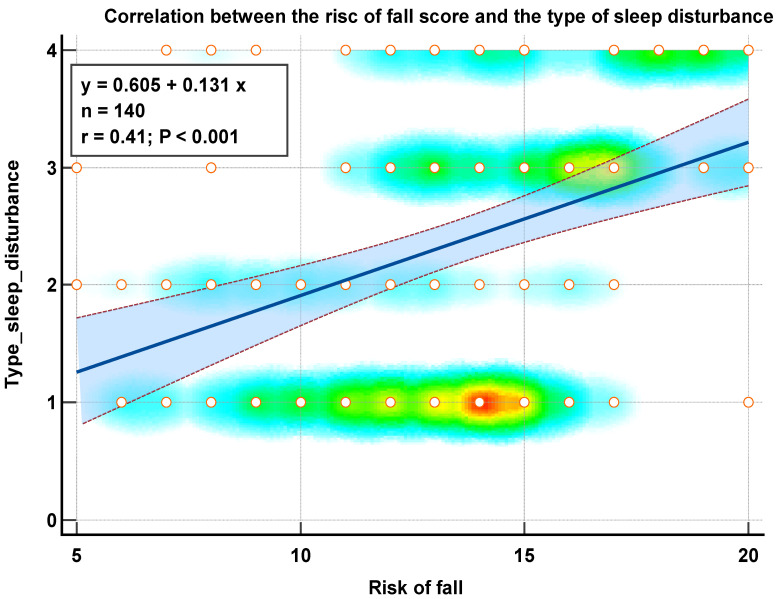
Correlation diagram between the type of sleep disturbance and the risk of fall score in the elderly.

**Figure 5:**
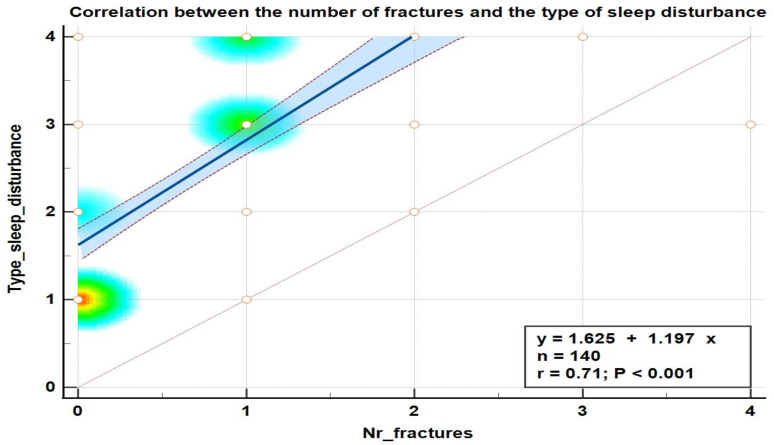
Correlation diagram between the type of sleep disturbance and the number of fractures in the elderly.

**Figure 6:**
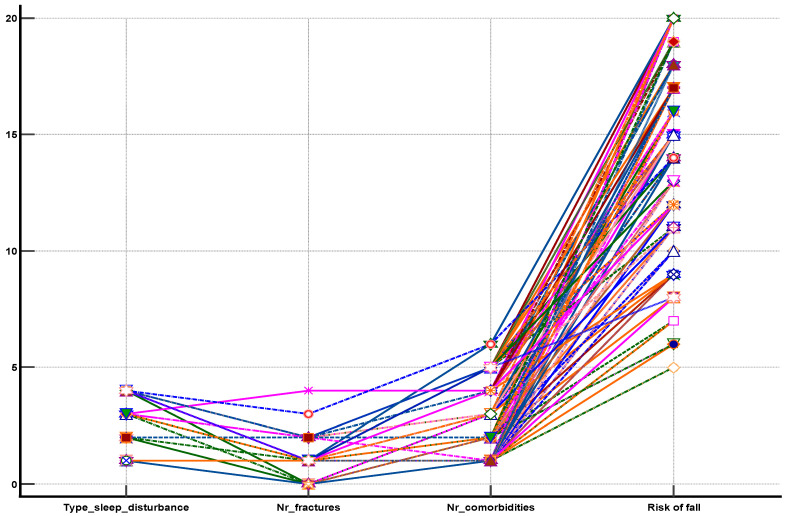
Diagram showing of the strong association between the sleep disturbances types, the number of comorbidities, the risk of fall score, and the number of fractures in the elderly.

We found positive and strong correlations between the type of sleep disturbance and the risk of fall (Figure 4), the type of sleep disturbance, and the number of fractures (Figure 5) as well as between the type of sleep disturbance, the number of comorbidities, the risk of fall score, and the number of fractures that occurred after the age of 65 years (Figure 6).

## 4. Discussion

The purpose of this study was to investigate the links between sleep problems (insomnia), comorbidities, multisite pain, physical activity, and fall risk in the elderly and to compare these parameters in elderly patients with and without fractures that occurred after the age of 65 years.

The study confirmed the study hypothesis regarding the significant association between insomnia of type 3 and 4, number of comorbidities, risk of fall, and the occurrence of fractures in patients aged ≥65 years.

Univariate logistic regression found that the number of comorbidities, the risk of fall, and types 1, 2, 3, and 4 of sleep disturbances (insomnia) were notably associated with the occurrence of fractures after the age of 65 years. Multivariate logistic regression analysis highlighted four independent variables associated with the occurrence of fractures, and these are the number of comorbidities (*p* = 0.0286, odds ratio 4.46, 95% CI 1.16–17.04), the risk of fall (*p* = 0.0058, odds ratio 4.91, 95% CI 1.04–23.17), sleep disturbance of type 3 (*p* = 0.0027, odds ratio 1.86, 95% CI 0.66–24.42), and sleep disturbance of type 4 (*p* = 0.001, odds ratio 4.88, 95% CI 1.02–23.40). The occurrence of fractures was significantly associated with a number of comorbidities > 2 and a risk of fall score > 14.

To our knowledge, this is the first study performed in Romania that evaluated the association between sleep disturbances and fractures in the elderly.

Physical activity is an important facilitator of a healthy lifestyle. According to the Active Ageing Index Report, Romania is among the European Union member states with the lowest scores, while the Nordic nations and the United Kingdom are at the top of the list [23].

Despite the health advantages of exercise, older persons are less likely to engage in physical activity. According to statistics, over 45% of adults aged 65 and up do not integrate exercise in their lifestyle, with physical inactivity being a serious worry for public health officials globally [24,25,26]. There are several intrapersonal, interpersonal, communal, and policy-based barriers that prohibit older persons from participating in physical activities [27,28].

Research suggests that older persons avoid sports activities owing to concerns about losing their balance and falling, muscular weakening, poor coordination, insufficient exercise capacity, and a lack of an appropriate activity [29,30]. In terms of emotions, research have shown that older persons are more prone to suffer from depression, which has a significant impact on their health. Moreover, the overlap between depression and anxiety as a twofold threat to older individuals’ quality of life should be considered [31,32,33,34,35]. Older persons prefer to minimize anxiety symptoms and explain them away by blaming them on physical limitations [36], avoiding physical activities as a result of this negative state.

Falls cause a significant amount of injury-related morbidity and death among the elderly, and the resulting fractures are a significant source of economic burden in this group [37]. Every year, one-third of people over the age of 65 and more than half of those over the age of 80 have at least one fall-related injury [38]. The significance of falls as a cause of senior trauma and the economic burden they impose have been demonstrated all over the world [39]. The prevalence of falls and the difficulties that come from them are increasing as the older population grows, and this trend will continue unless steps are taken to enhance the services provided to vulnerable groups.

Our findings show that there is an independent relationship between sleep disorder and fracture risk in the elderly. We discovered that having a type 3 or 4 sleep disturbance was not only related with a higher falls-risk score but also increased the likelihood of any fracture, and this association was irrespective of comorbidities and BMD.

Prior research on the links between sleep issues and falls has produced inconsistent results, with some studies indicating reduced BMD in adults with sleep disorders [14,15,40] and others reporting no relationships [16,17,18]. Our current findings are in line with the results of previous cross-sectional studies that demonstrated no link between sleep disorders and BMD. In our investigation, the elderly with fractures had lower total BMD, although the difference was not statistically significant. We discovered that the elderly with fractures had notably more comorbidities, more serious sleeping problems, and a higher risk of falling.

This study’s findings that sleep disruption was related with a higher falls-risk score were consistent with earlier research that examined the association between sleeping disorders and self-reported falls. In a prospective study of postmenopausal women, self-reported sleep parameters such as sleep quality and duration were linked to two or more falls during each year of follow-up [41]. Similarly, Stone et al. [42] discovered that self-reported short sleep and daily napping were related with an increased risk of having two or more falls in the following year among community-dwelling older women. Their findings were later confirmed in a study using objective measures (i.e., actigraphy) to assess sleep problems in the same population and in a community-dwelling group of older men [43]; they discovered that those who slept 5 h per night had a higher risk of suffering two or more falls over a 1-year follow-up than those who slept 7–8 h per night. Gassmann et al. [44] discovered that sleep disruption was a significant predictor of occasional and recurrent falls in the previous six months in older persons.

Our research builds on previous studies by employing an objective and complete evaluation that can detect an individual’s risk of falling. In this study, an objectively assessed falls-risk score, as opposed to self-reported falls, allowed for the early identification of persons at high risk of falling. Given the complex nature of falls, the findings of this study and other research indicate that screening patients with sleep issues for intervention/treatment (e.g., cognitive behavioral therapy) may protect against falls. Nevertheless, the extent of an increase in falls-risk score as a result of sleep disruption that translates into falls incidence is unknown. We found a strong positive correlation between the severity of sleep disorders in the elderly and the risk of fall. We also demonstrated that a risk of fall score > 14 was independently associated with fractures in the elderly. A sleep disturbance of type 3 doubled the risk of fractures, while a sleep disturbance of type 4 increased this risk by five. These findings imply that improving sleep may be able to minimize falls and fractures in the elderly, although the underlying processes of these associations remain unclear.

Effective fall prevention can reduce serious fall-related injuries, emergency department visits, hospitalizations, nursing home placements, and functional decline. One of the most fundamental elements to consider while providing services to senior patients suffering from fall-related injuries is service quality, with an emphasis on proper patient evaluation and identification of risk factors that increase susceptibility to falls. Identification of at-risk patients and treatments to mitigate this risk should become an essential element of the elderly-assessment process.

We will perform future research to explore the relationship between fall risk, sleep disorders, and functionality. In this respect, we will apply multicomponent training, which consists of the exercise pattern warm-up, aerobic activity, muscle strengthening, and relaxing. This exercise program has proven to be the best for the aged population to improve their muscle power and functional ability [45].

### Study Limitations 

The fact that this study had a small number of participants has an impact on the conclusions that can be drawn. The strategy used to recruit participants may have resulted in a possible research bias, as the older persons in this study were recruited from nursing homes for the aged. Examining persons from rural regions also might enrich future analyses of Romanian older adults’ lifestyle habits. Sleep quality was assessed using a self-reported questionnaire, which might have resulted in recollection bias. All patients with sleep disturbances of type 3 and 4 received medication for insomnia (non-benzodiazepine hypnotics), and this medication could induce dizziness and muscular weakness. The same effect could have been induced by the medication for comorbidities, which was included in the calculation scale of the risk of fall.

## 5. Conclusions

A fall-risk score over 14 and a number of comorbidities over 2 were notably associated with the occurrence of fractures. We also found strong positive correlations between the type of sleep disturbance, the risk of fall score, the number of comorbidities, and the number of fractures in the elderly. Further study is needed to validate and understand the processes underlying these correlations and to find adequate methods to decrease the risk of falls/fractures in the elderly.

## Figures and Tables

**Table 1:** Patients’ characteristics by the presence of fractures.

Parameter	Group I (Without Fractures) *n* = 67	Group II (With Fractures) *n* = 71	*p*-Value
Age, years (X ± DS)	78.2 ± 2.5	78.9 ± 2.3	0.08
Male gender, *n* (%)	29 (43%)	26 (37%)	0.47
Body mass index, kg/m^2^	28.39 ± 5.14	29.51 ± 6.19	0.25
Obesity, *n* (%)	26 (39%)	17 (24%)	0.05
Smoker, *n* (%)	36 (54%)	37 (52%)	0.81
Comorbidities, number (X ± SD)	1.47 ± 0.63	3.73 ± 1.44	<0.0001
Number steps/day, (X ± SD)	2424.67 ± 1653.91	2933.75 ± 1610.24	0.06
Multisite pain, *n* (%)	34 (51%)	28 (39%)	0.15
Total bone mineral density (g/cm^2^)	2.36 ± 0.72	2.27 ± 0.64	0.26
Risk of fall, (X ± SD)	11.31 ± 3.12	15.63 ± 2.99	<0.0001
Number of fractures, (X ± SD)	0	1.15 ± 0.33	-
Sleep disturbances, *n* (%)			
Type 1	18 (27%)	8 (11%)	0.01
Type 2	20 (30%)	10 (14%)	0.02
Type 3	14 (21%)	26 (37%)	0.03
Type 4	15 (22%)	27 (38%)	0.04
Medication for sleep disturbances			
Zopiclone	29 (43%)	41 (75%)	<0.001

Chi-square tests were used to compare categorical data and Student’s *t*-tests to compare continuous data. Abbreviations: X, arithmetic mean; SD, standard deviation; *n*, number.

**Table 2:** Parameters associated with the occurrence of fractures in the elderly in univariate and multivariate logistic regression.

Parameter	Univariate Analysis OR (95% CI)	*p*-Value	Multivariate Analysis OR (95% CI)	*p*-Value
Number of comorbidities	4.90 (2.95 to 8.16)	<0.0001	4.46 (1.16 to 17.04)	0.0286
Risk of fall score	8.97 (4.38–18.36)	<0.0001	4.91 (1.04 to 23.17)	0.0058
Sleep disturbance type 1	0.02 (0.00 to 0.08)	<0.0001	-	-
Sleep disturbance type 2	0.07 (0.0 to 0.35)	<0.0001	-	-
Sleep disturbance type 3	6.24 (1.82 to 47.44)	<0.0001	1.86 (0.66 to 25.42)	0.0027
Sleep disturbance type 4	5.28 (1.69 to 40.15)	<0.0001	4.88 (1.02 to 23.40)	0.0010

Abbreviations: OR, odds ratio; CI, confidence interval.

## Data Availability

No supplementary data are available.
